# Social Factors of Dietary Risk Behavior in Older German Adults: Results of a Multivariable Analysis

**DOI:** 10.3390/nu14051057

**Published:** 2022-03-02

**Authors:** Christoph Geigl, Julika Loss, Michael Leitzmann, Christian Janssen

**Affiliations:** 1 Department of Applied Social Sciences, Munich University of Applied Sciences, 81243 Munich, Germany; 2 Department of Epidemiology and Preventive Medicine, University of Regensburg, 93053 Regensburg, Germany; michael.leitzmann@klinik.uni-regensburg.de; 3 Department of Epidemiology and Health Monitoring, Robert Koch Institute, 13353 Berlin, Germany; lossj@rki.de

**Keywords:** dietary risk behavior, older age, older German adults, social factors, gender, socioeconomic status, multiple linear regression analysis, principal component analysis

## Abstract

With this analysis, we aimed to examine the associations between social factors and dietary risk behavior in older adults. Data were collected through a full-population postal survey of German adults aged 65 years or older (*n* = 1687, 33% response proportion, 52% female, mean age = 76 years). Using principal component analysis (PCA), a data-driven Dietary Risk Behavior Index (DRB) was computed. Dietary risk behavior was defined as consumption frequencies of vegetables/fruit, whole grains, and dairy products below national dietary recommendations. By performing a multiple linear regression, we analyzed associations between sociodemographic, socioeconomic, psychosocial, and behavioral factors and dietary risk behavior. Physical activity, female gender, socioeconomic status, social support, and age (in the male sample) were negatively associated with dietary risk behavior. Alcohol consumption and smoking were positively associated with dietary risk behavior. A group-specific analysis revealed a higher goodness-of-fit for the low socioeconomic status group, older adults aged 65–79 years, and women. A comprehensive understanding of the relationships between social factors and dietary risk behavior in older adults assists the group-specific targeting of dietary-related interventions. Demand-oriented dietary interventions should account for underlying social conditions to reduce inequity in dietary risk behavior among older adults. The results of this work may be transferable to municipalities in high-income European countries.

## 1. Introduction

Dietary behavior has an important impact on the burden of non-communicable diseases (NCDs) [1,2,3,4]. In 2017, NCDs such as cardiovascular diseases, cancers, and diabetes accounted for 11 million diet-related deaths [5]. In addition, 255 million attributable disability-adjusted life years (DALYs) [6] could be traced back to dietary risk factors. DALY is a widely accepted measure combining the time lived with disability and/or illness with time lost due to premature death [7].

In the Global Burden of Disease Study (GBD) 2017 [7], dietary risks were the dominant level 2 risk factor for deaths and the second-most common level 2 risk factor for DALYs. Level 2 of the GBD includes more specific categories for deaths and DALYs such as NCDs. The number of diet-related deaths and DALYs showed an increasing trend by age. Deaths peaked at age 70–74 years for males and age 80–84 years for females; DALYs peaked for both gender groups at age 65–69 years [8]. According to the GBD 2017 [5], improving dietary behavior across nations could prevent one in five deaths worldwide. Among the globally leading dietary risk factors are low intakes of vegetables, fruit, whole grains, and dairy products [5,9,10]. At the global level, the consumption of these food groups is below the theoretical minimum risk exposure level (TMREL) that minimizes the risk from all causes of death [5]. Diets lacking in vegetables (<290–430 g per day), fruit (<200–300 g per day), whole grains (<100–150 g per day), and dairy products (<350–520 g per day) cause 65% of all diet-related deaths and 72% of all diet-related DALYs [7].

Globally, the number and proportion of adults aged 65 years or older are increasing. In 2020, the number of people over 65 years of age was estimated to be 727 million and is forecast to double to around 1.5 billion by 2050. Hence, the proportion of the population aged 65 years or older is predicted to rise by 9%–16%, meaning one in six people will be aged 65 years and above [11]. As the population ageing process continues, the prevalence of chronic diseases and multimorbidity will also increase [12,13]. For older adults, NCDs are among the predominant health risks [14].

In studying dietary behavior, the analysis of dietary patterns has been accepted as a complementary approach to the analysis of single food groups. Analysis of dietary patterns is likely to be more appropriate for predicting trends in dietary behavior than analysis of single food groups [15,16,17], while still being suitable for referring to dietary recommendations at the level of single food groups [18]. There are two main approaches to assessing dietary patterns: first, the data-driven approach (a posteriori), reducing the dimensionality of food groups while preserving as much variance as possible; second, the hypothesis-driven approach (a priori), using pre-defined scoring systems conventionally based on dietary recommendations [19]. In this work, we used a combination of both to create a data-driven Dietary Risk Behavior Index (DRB) relating to consumption frequency recommendations of vegetables/fruit, whole grains, and dairy products.

Dietary behavior is one of the most important predictors of health [9,10], which is potentially modifiable. An impactful approach to improving dietary behavior could be achieved by considering social conditions [20,21,22]. Among older adults, several studies have examined the relationship between social factors and dietary patterns. Analyzed dietary patterns vary widely, but also include patterns based on vegetables, fruit, whole grains, and dairy products [19,23,24,25,26]. However, the relationship between social factors and dietary patterns has not been thoroughly investigated to date [22,27,28,29].

The impact of age on dietary behavior among already older adults is ambiguous, as the results of previous research are partly inconsistent. While age was negatively associated with a “vegetable-based” pattern [30], no associations were observed between age and a “Mediterranean” pattern [19], or age and a “healthy” pattern [31]. In the analysis of age groups, a “healthy” pattern was negatively associated with the 75 years or older age group in the female sample, whereas no association could be found for the 70–74 age group [24].

Concerning gender, older women were constantly associated with a higher consumption of vegetables, fruit, whole grains, and dairy products [19,24,30,31,32]. Dietary patterns have often been analyzed in the context of living arrangements (alone/married, couple or cohabitation). Some studies have been able to show a negative association between living in a couple or cohabitation and “healthy” patterns [24,33], while others have found no association between living arrangements and “healthy” [33] or “Mediterranean” [19] patterns. We analyzed the relationship between partnership status and dietary risk behavior status. Partnership status indicates whether respondents live in a partnership or not. We defined a partnership as a married couple or a couple without being married.

As for education, several studies have provided evidence linking higher educational levels for older adults with a higher intake of vegetables, fruit, whole grains, and dairy products [19,24,31,32,33]. The role of income in the dietary patterns of older adults is largely unknown. There are relatively few studies that have reported findings on the relationship between income and dietary patterns. In two cohorts, no association was found between income and a “healthy” pattern [33]. However, in another cohort, income was positively associated with a “healthy” dietary pattern.

Thus far, the influence of health-related orientations such as the health locus of control on dietary behavior has not been determined [34]. From the few available studies, hardly any conclusions can be drawn [35,36]. However, associations with behavioral factors may indicate a confounding effect of the health locus of control [36,37]. In terms of social support, some studies have found a link between social support and dietary behavior [22,38], but this relationship has yet to be thoroughly investigated in older adults. It should be noted that there is a positive association between social support and physical activity in older adults [39], suggesting a confounder effect of social support.

A few studies among older adults have demonstrated that higher physical activity [19,30,31] and non-smoking status [24,31,33] are associated with a higher prevalence of dietary patterns based on vegetables, fruit, whole grains, and dairy products [19]. There is limited evidence on the relationship between alcohol consumption and dietary behavior in older adults, with inconclusive results. Thus, positive associations between alcohol consumption and inadequate vegetable and fruit intake [40], or non-consumers and the highest vegetable and fruit intake, were found [41]. Another study, however, could not show any associations between alcohol consumption and vegetables, fruit, whole grain, and dairy products [42].

To date, an in-depth analysis of the relationship between social factors and the dietary risk behavior of older adults in Germany is still pending. What is principally missing is an understanding of the structural relationship and impact levels between social factors and dietary risk behavior in older age. It is unclear how social factors interact with dietary risk behavior and which social factors are more associated with dietary risk behavior than others. Moreover, the influences of age, income, partnership status, health locus of control, social support, and alcohol consumption on dietary behavior in older age still appear undetermined.

Hence, the objective of the present work was to analyze the relationship between sociodemographic, socioeconomic, psychosocial, and behavioral factors and dietary risk behavior among older adults. We used data from a sample of German adults aged 65 years or older to examine social factors and dietary risk behavior in a comprehensive statistical model. Our findings can serve as a basis for specific dietary interventions to decrease inequity in dietary risk behavior in older adults.

The present analysis aimed to examine:

(1) how many older adults are affected by dietary risk behavior related to low consumption frequencies of vegetables/fruit, whole grains, and dairy products;
(2) differences in dietary risk behavior in older age, stratified by gender, age, and socioeconomic status groups;
(3) differences in consumption frequencies of vegetables/fruit, whole grains, and dairy products among older adults stratified by gender, age, and socioeconomic status groups;
(4) associations between sociodemographic, socioeconomic, psychosocial, and behavioral factors and dietary risk behavior in older age; and
(5) differences in the statistical model stratified by gender, age, and socioeconomic status groups.

## 2. Materials and Methods

### 2.1. Data and Sample

The analysis was based on data from the Healthy Municipality Puchheim project conducted by the Munich University of Applied Sciences (HM) in 2019. Cross-sectional data were collected by a full-population postal survey of all residents aged 65 years or older in Puchheim, Germany. The address data of the population were provided by the municipal administration and the residents’ registration office. The entire study population consisted of 5102 older adults, of whom 1687 (33%) returned the questionnaire. All participants received a standardized self-administrated questionnaire (SAQ) with a letter from the principal mayor and the HM project manager.

The study was approved by the HM Ethics Committee (EK006HM–03_21) and strictly adhered to German data safety regulations.

### 2.2. Measures

A simplified Food Frequency Questionnaire (FFQ) [43] was assessed to indicate the frequencies of consumption of nine food groups, including vegetables/fruit, whole grains, and dairy products. Each item can take on values ranging from 1 to 6 points, with higher values stating a higher frequency of consumption. In 2020, the German Nutrition Society (DGE) [44] specified the minimum consumption frequencies of vegetables (three times a day), fruit (two times a day), whole grains (two times a day), and dairy products (two times a day) for older adults, which can be considered as dietary risk behavior if not reached. This recommendation was met in our analysis if respondents reported consuming each food group several times a day; once a day was not sufficient. For further analysis, a brief Dietary Risk Behavior Index (DRB) was calculated as a weighted component score, including the food groups comprising vegetables/fruit, whole grains, and dairy products. The DRB took on values between −2 and 3 points in our sample, and higher values indicated higher dietary risk behavior. The standardized DRB has a mean of 0 and a standard deviation (SD) of 1.

The Socioeconomic Status Index (SES) [45,46] was determined as a sum score based on three-point scores for educational level, (previous) occupational status, and equivalized disposable income. For categorization into three SES groups (low/middle/high), a distribution-based delineation into quintiles was carried out, with the three middle groups (second to fourth quintiles) combined. Educational level and income values ranged between 1 and 7 points each, with higher values reflecting a formally higher education and a higher income. The use of occupational status as an independent variable was dismissed due to its low variance.

The Health Locus of Control Scale (HLC) [47] was applied to rate a person’s level of belief that health and illness are controlled internally or externally. Example items include “I am directly responsible for my health” (internal) and “Doctors control my health” (external). The response categories were given as five-point Likert scales on which respondents could indicate their agreement or disagreement with the respective items. Internal (Cronbach’s alpha of 0.8) and external (Cronbach’s alpha of 0.6) HLC were calculated as unweighted sum scores ranging from 1 to 5 points (Cronbach’s alpha of 0.6). The unidimensional HLC was calculated by subtracting the external score from the internal score. The HLC took on values between −3 and 3 points in our sample, and higher values correspond to more pronounced internal control tendencies.

Social support was measured using the valid Oslo 3-Item Social Support Scale (OSS-3) [48]. The OSS-3 refers to the number of people respondents feel close to, the interest and concern showed by others, and the ease of obtaining practical help from others. A sum score was calculated by summarizing the valid values of the three items used. OSS-3 can take on values between 3 and 14 points (Cronbach’s alpha of 0.7), with higher values representing stronger levels of social support [49].

The three-item Alcohol Use Disorder Identification Test-Consumption (AUDIT-C) [50] is a validated brief screening instrument and was used to assess risky alcohol consumption. The AUDIT-C was constructed by adding the valid values of the three items related to a sum score. Values of the sum score can range from 0 to 12 points, with a maximum of 11 points in our sample. Higher values suggest riskier alcohol consumption behavior.

Physical activity (PA) [43] was calculated as an unweighted sum score based on the frequencies of sports and more physically demanding daily activities. Both items can take on values ranging from 1 to 6 points. Thus, the value of the sum score ranges from 2 to 12 points, and higher values indicate higher physical activity.

Smoking status was coded as a dichotomous risk behavior (current smoker/non-smoker). Daily and occasional smokers were grouped as current smokers, while ex-smokers and never-smokers were categorized as non-smokers [51].

Age was calculated from the year of birth. For stratification, two groups of 65–79- and 80–98-year-olds were formed. Although diversity in gender identities cannot be captured with binary response options [52], we used the term “gender” to emphasize socially constructed differences that go beyond biological sex [53]. Partnership status indicates whether respondents were in a partnership or not. The required information was collected according to national demographic standards [54].

### 2.3. Statistical Analysis

The sample was analyzed by calculating the absolute and relative frequencies for sociodemographic variables and the consumption frequencies of the single food groups.

Pearson’s chi-squared test (*χ*²) was used to examine differences in dichotomous variables of dietary risk behavior (risk/no risk) stratified by gender (male/female), age (65–79 years/80–98 years), and SES groups (low/middle/high).

Mean values of the food group variables were stratified by gender, age, and SES groups. For comparing the mean differences in gender and age groups, the *t*-test was used, and for SES groups, we employed the one-factor analysis of variance (ANOVA). Levene’s test was used to assess the homogeneity of variance when the *t*-test and ANOVA were carried out. If the *p*-value was significant, Welch adjustments were assessed.

Principal component analysis (PCA) was performed to reduce the dimensionality of the food groups while retaining most of the variance in the data. The data set was approved for suitability using Bartlett’s test of sphericity and the Kaiser–Meyer–Olkin test. To improve the interpretability of the components and to achieve a more even distribution of variance, the component rotation varimax was utilized. A data-driven dietary pattern centered on the food groups of vegetables/fruit, whole grains, and dairy products could be extracted (a posteriori). From the main food groups of the DP extracted, a brief Dietary Risk Behavior Index (DRB) was created. Therefore, a weighted sum score was calculated by adding the inverse-coded scores of the single food group consumption frequencies. Dietary risk behavior was defined based on current national dietary recommendations [44] as the insufficient consumption of vegetables, fruit, whole grains, and dairy products (a priori). Thus, the sum score of the DRB reflects the extent of underconsumption of these food groups. For reliability analysis, Cronbach’s alpha was calculated to assess the internal consistency of the index.

Missing values analysis for variables used in regression analysis was initially based on univariate statistics. Group differences between valid and missing values were analyzed using the *t*-test. All missing values of the variables used in the regression analysis were estimated by multiple imputation. The number of imputation data sets to compute was specified as five (m = 5). Thus, the output dataset consists of the original case data plus five complete data sets with imputed values in each case. The automatic method was used as the imputation method, and the allowable range of imputed values was restricted to plausible values (minimum, maximum, rounding). In the regression analysis, outputs were generated for the original data set, each of the five complete data sets, and a pooled output.

Multiple linear regression was performed to analyze the associations of social factors and the DRB. Social factors were introduced according to a predefined structure using a hierarchical method. Adjusted *R*^2^ values were interpreted as a measure of goodness-of-fit, according to Cohen [55], and *R*^2^ was used to report the explained variance. In line with evidence of differences in dietary patterns by gender [26,56], age groups [24,57], and socioeconomic status groups [58,59], we performed tests for interaction between these groups. We then created gender-, age group-, and socioeconomic status group-specific models to analyze potential differences. All requirements for regression analysis [60] were proved and considered valid. Heteroscedasticity-consistent standard error estimators (HC4) were routinely applied to ensure validity and power [61].

Statistical analysis was conducted with IBM^®^ SPSS^®^ Statistics software (version 27.0.0.0). All statistical significances were defined as *p* < 0.05.

## 3. Results

### 3.1. Participants

The sample consisted of 1687 older adults with an average age of 76.4 years (range 65–98 years). A group of 1116 respondents (68%) were aged 65–79 years, and a group of 519 respondents were aged 80 years or older. The nationality of the vast majority (97%) was German.

Descriptive statistics of the variables used in the regression analysis are displayed in Table 1. Higher scores for the metric variables stand for a stronger expression. Mean values of gender, partnership status, and smoking status variables can be interpreted as a percentage of the distribution.

We identified missing values of 0.8–10% within each variable (Table 1). Analysis of missing values showed that respondents who did not report income (*p* = 0.03), alcohol use (*p* < 0.001), and dietary risk behavior (*p* < 0.001) were older on average.

### 3.2. Frequencies of Consumption and Dietary Risk Behavior

The consumption frequencies of vegetables/fruit, whole grains, and dairy products are presented in Table 2. The percentages of the consumption frequency refer to the valid data. National dietary recommendations [44] were met if the respondents reported consuming each food group several times a day; once a day was not sufficient. Minimum frequencies of consumption were not achieved for vegetables/fruit by 1096 older adults (67%), whole grains by 1452 older adults (91%), and dairy products by 1307 older adults (81%). Overall, only 51 older adults (3%) met the minimum consumption frequency across all three food groups.

### 3.3. Sociodemographic and Socioeconomic Differences in Dietary Risk Behavior

Consumption frequencies for vegetables/fruit, whole grains, and dairy products below the national recommendations were assessed as dietary risk behavior. On average, men showed higher dietary risk behavior than women in the consumption frequencies of vegetables/fruit (*p* < 0.001, Φ = −0.2), whole grains (*p* = 0.03, Φ = −0.1), and dairy products (*p* < 0.001, Φ = −0.1). All the effect sizes ranged from small to medium [55] (Table 3).

### 3.4. Sociodemographic and Socioeconomic Differences in Consumption Frequencies

Compared to women, men consumed fewer vegetables/fruit (*p* < 0.001, d = 0.4), whole grains (*p* < 0.001, d = 0.2), and dairy products (*p* < 0.001, d = 0.3) on average. Lower SES groups consumed fewer vegetables/fruit (*p* = 0.001, η² = 0.01) and dairy products (*p* = 0.003, η² = 0.01) than higher SES groups on average. All effect sizes were in the small-to-medium range [55] (Table 4).

### 3.5. Dietary Risk Behavior Index

The Kaiser–Meyer–Olkin measure of sampling adequacy (MSA) was 0.6 (mediocre) [62] and the *p*-value of Bartlett’s test for sphericity was significant (*p* < 0.001). The anti-image correlation values ranged from 0.56 to 0.63. Thus, the data were adequate for PCA [60]. A dietary pattern of vegetables/fruit, whole grains, and dairy products could be extracted. Communalities (≥0.5) and component loadings (≥0.7) fulfilled the statistical requirements [63] (Table 5). The Dietary Risk Behavior Index (DRB) represented 51.34% of the total variance in the food groups included. The internal consistency of the index was relatively low (Cronbach’s alpha of 0.51).

Analysis of the DRB showed that men consumed more riskily than women (*p* < 0.001, d = 0.42), and lower SES groups consumed more riskily than higher SES groups on average (*p* < 0.001, η² = 0.01). All effect sizes ranged from small to medium [55]. There was no statistically significant difference in dietary risk behavior between the age groups of 65–79 and 80–98 years (*p* = 0.58).

### 3.6. Associations between Social Factors and Dietary Risk Behavior

The results of the multiple linear regression of the four models in which the variables were included in a hierarchical structure are reported in Table 6. In the first model, which explained 4% of the variance in dietary risk behavior (*R*^2^ = 0.04, Adj. *R*^2^ = 0.04, *F*(3, 1683) = 22.61, *p* < 0.001), female gender decreased dietary risk behavior, while age and partnership status had no significant effect. As the socioeconomic factors were introduced, the explained variance of dietary risk behavior increased to 8% in the second model (*R*^2^ = 0.08, Adj. *R*^2^ = 0.07, *F*(5, 1681) = 27.92, *p* < 0.001). Educational level was negatively associated with dietary risk behavior, and the coefficient of female gender increased. Income had no significant effect. In the third model, which slightly increased the explained variance to 9% (*R*^2^ = 0.09, Adj. *R*^2^ = 0.08, *F*(7, 1679) = 22.29, *p* < 0.001), psychosocial factors were introduced. The coefficient of female gender marginally increased, while the educational level coefficient decreased. The health locus of control and social support were negatively associated with dietary risk behavior. With the introduction of behavioral factors, the explained variance considerably increased to 16% in the fourth model (*R*^2^ = 0.16, Adj. *R*^2^ = 0.16, *F*(10, 1676) = 32.48, *p* < 0.001). Female gender and educational level decreased the coefficient size. The health locus of control lost its significant effect. The coefficient of social support decreased slightly. Smoking status and alcohol use were positively associated with dietary risk behavior, whereas physical activity was negatively associated. Each block of introduced variables led to a significant improvement in the goodness-of-fit (*p* < 0.001). Adjusted *R*^2^ for the final model indicated a moderate goodness-of-fit [55]. In terms of the final model and its variables, physical activity had the largest coefficient, followed by female gender, alcohol use, and educational level. Smoking status and social support had the lowest coefficients.

When we analyzed the original data set (*n* = 1272) without multiple imputed values, age was negatively associated with dietary risk behavior in the second (β = −0.05; *p* < 0.04), third (β = −0.07; *p* < 0.005), and fourth (β = −0.07; *p* < 0.008) models.

The results of the analysis of the final model stratified by gender are displayed in Table 7. The model explained 13% of the variance in dietary risk behavior when analyzed for the male sample (*R*^2^ = 0.13, Adj. *R*^2^ = 0.12, *F*(9, 792) = 12.73, *p* < 0.001). Physical activity, educational level, and age were negatively associated with dietary risk behavior, while alcohol use was positively associated. When analyzed for the female sample, the model explained 15% of the variance (*R*^2^ = 0.15, Adj. *R*^2^ = 0.14, *F*(9, 875) = 16.95, *p* < 0.001). Physical activity, educational level, and social support were negatively associated with dietary risk behavior, whereas alcohol use was positively associated. Tests for interactions by gender showed significant effects for age (*p* = 0.02), partnership status (*p* = 0.02), and physical activity (*p* = 0.04). Goodness-of-fit was higher for the sample of females than for the males.

The results from the analysis of the final model stratified by age groups are shown in Table 8. The model explained 18% of the variance in dietary risk behavior in the sample of older adults aged 65–79 years (*R*^2^ = 0.18, Adj. *R*^2^ = 0.18, *F*(9, 1131) = 28.52, *p* < 0.001). Physical activity, female gender, educational level, and social support were negatively associated with dietary risk behavior, while alcohol use and smoking were positively associated. In the sample aged 80–98 years, the model explained 12% of the variance (*R*^2^ = 0.12, Adj. *R*^2^ = 0.11, *F*(9, 536) = 8.55, *p* < 0.001). Physical activity, female gender, and educational level were negatively associated, while alcohol use was positively associated. Tests for interactions by age groups showed no significant effects. Goodness-of-fit was higher for the age group of 65–79 years than for the age group of 80–98 years.

The analysis of the model stratified by SES groups is presented in Table 9. For the low SES group, the model explained 25% of the variance in dietary risk behavior (*R*^2^ = 0.25, Adj. *R*^2^ = 0.23, *F*(8, 339) = 13.81, *p* < 0.001). Physical activity, female gender, and social support were negatively associated with dietary risk behavior, whereas alcohol use was positively associated. In the middle SES group, the model explained 13% of the variance (*R*^2^ = 0.13, Adj. *R*^2^ = 0.12, *F*(8, 957) = 17.34, *p* < 0.001). Physical activity and female gender were negatively associated with dietary risk behavior, while alcohol use was positively associated. In the high SES group, the model explained 11% of the variance (*R*^2^ = 0.11, Adj. *R*^2^ = 0.09, *F*(8, 364) = 5.29, *p* < 0.001) and physical activity was negatively associated. Tests for interactions by socioeconomic status groups showed an significant effect for alcohol use (*p* = 0.04). Goodness-of-fit was higher for the lower SES groups than for the higher SES groups.

## 4. Discussion

### 4.1. Principal Results of the Analysis

The results of the analysis showed the importance of social factors regarding dietary risk behavior among older German adults. Physical activity, female gender, socioeconomic status, social support, and age (in the male sample) were negatively associated with dietary risk behavior, whereas alcohol use and smoking were positively associated. The health locus of control had no significant effect. Group-specific analysis revealed higher goodness-of-fit for the low SES group, older adults aged 65–79 years, and women, suggesting higher explained variance in dietary risk behavior in these groups. Our analysis provides a comprehensive understanding of the relationships between social factors and dietary risk behavior, which can be useful for developing diet-related interventions in specific groups. Social factors hold significant potential for improving dietary behavior through demand-orientated interventions, considering different social conditions appropriately.

Most of the respondents did not comply with the national minimum intake requirements for the single food groups of vegetables/fruit, whole grains, and dairy products. Men were more likely to fail to meet the minimum consumption frequencies of the single food groups than women. On average, men consumed fewer vegetables/fruit, whole grains, and dairy products than women, and lower SES groups consumed fewer of these food groups than higher SES groups. We were able to demonstrate the same trends for the dietary risk behavior index. The reported findings are of interest as they highlight the need to uncover the various components of the complex relationship between social circumstances and dietary risk behavior among older adults. Variability in dietary behavior among the elderly suggests that some older adults are more able to adhere to the minimum consumption frequencies, while others are not.

### 4.2. Strengths and Limitations

A major strength of this analysis is the large, up-to-date data set collected through a full-population survey of older German adults, including essential social and dietary factors. Thus, a more in-depth analysis of the associations between social factors and dietary risk behavior could be conducted in this specific age group. Due to the size of the sample, a separate analysis of different gender, age, and SES groups could be performed, which provided more detailed insights. To the best of our knowledge, this is the first analysis in a comprehensive statistical model examining the relationships of sociodemographic, socioeconomic, psychosocial, and behavioral factors with dietary risk behavior relating to vegetables, fruit, whole grains, and dairy products. This is surprising, as the impact of a diet low in vegetables, fruit, whole grains, and dairy products on DALYs and death rates has been impressively demonstrated by the Global Burden of Disease Studies [5,9,10]. As one of the few analyses of dietary patterns, our analysis considered the underlying structure of social factors and attempted to formulate an explanatory approach to the structure of the social factors associated with dietary risk behavior. Through comprehensive statistical model analysis, the relationships between different social factors and dietary risk behaviors can be weighed against one another, enabling more efficient and effective targeting of dietary interventions [60]. A brief Dietary Risk Behavior Index (DRB) consisting of fewer single food groups increases the reproducibility and comparability of the results. A brief food frequency questionnaire (FFQ) of the index food groups can be accommodated in health surveys in a resource-efficient manner, in contrast to the usually extensive FFQs.

However, the analysis also has several limitations, so the presented results should be interpreted carefully. Based on the cross-sectional data set, no conclusions can be drawn about causal relationships. Self-reported data sets are inherently biased for many reasons. Some subgroups of the population are usually underrepresented in data sets, including institutionalized older adults, older adults with a lower SES, or older adults with disabilities or diseases [64]. In our analysis, we examined older adults of predominantly German citizenship. Residents with other citizenships could rarely be reached. Hence, associations between social factors and dietary risk behavior may be underestimated in these subgroups. This is conceivable, especially for the influence of the health locus of control and age. Additionally, the dietary behavior and socioeconomic status data may have been subjected to social desirability bias. Lower SES groups, as well as older adults with disabilities or diseases, could have been more affected, leading to an underestimation of associations.

Related to our statistical model, we presume that our empirical model has model specification errors affecting model validity. We assume that additional factors such as health conditions [65], living environment [66], or psychological distress [67] may increase predictive model validity. Factors with low regression coefficients such as age, partnership status and health locus of control reduced the predictive validity of the model. The DRB based on vegetables/fruit, whole grains, and dairy products has proven to be useful for analyzing our data, but cannot be considered sufficiently validated. A weighted calculation of the index provides more precise results in our sample, since the food groups are included according to their component loading. Unfortunately, this approach complicates the interpretation of the index value and does not allow direct comparisons with other studies. To permit cross-study comparisons, an unweighted score-based index could be built upon for further analysis. For a relative assessment, cut-off points could be defined for low, moderate, and high dietary risk behavior. Frequencies of consumed food groups were assessed using a simplified nine-item FFQ. Of course, the extracted dietary pattern refers to the food groups used and our study population. Further studies are needed to explore the potential of the DRB to detect dietary risk behavior in a range of older populations.

### 4.3. Comparison with Other Studies

The role of age in the consumption of vegetables, fruit, whole grains, and dairy products is largely inconsistent, reflecting different dependent variables (single food groups and dietary patterns) in the analyses. In the EPIC study of older adults from nine European countries, aged 60 years or older, age was negatively associated with the “vegetable-based” pattern, consisting mainly of vegetables, fruit, and grains [30]. Among French adults aged 60 years or older in the NutriNet-Santé cohort, the “healthy” pattern, including vegetables, fruit, whole grain products, nuts, and fish, was negatively associated with the age group of 75 years or older in the female sample. However, no association could be found for the age group of 70–74 years [24]. No association was observed between age and the “Mediterranean” pattern, including vegetables, fruit, and dairy products, in older New Zealand adults aged 65–74 years (REACH study) [19] and the “healthy” pattern based on vegetables, fruit, and legumes in older American adults aged 65 years or older (HRS and HCNS) [31]. In our analysis, age was negatively associated with dietary risk behavior in the male sample. Although this does not mean that age does not influence dietary risk behavior in other subgroups, indirect contributions are also possible. Further research would be of interest to understand the role of age in dietary behavior in older adults [19].

In terms of gender roles, older women have constantly been associated with the “healthy” and “Mediterranean” patterns described above [19,24,31]. This is consistent with our finding that women tended to consume more vegetables/fruit, whole grains, and dairy products and were less likely to engage in dietary risk behavior than men on average. In the regression analysis, gender was examined using two common approaches to ensure a gender-sensitive procedure. Firstly, gender was analyzed as a predictor in the regression models; secondly, two models were analyzed separately for each gender. When we analyzed gender as a predictor, the male gender was associated with higher dietary risk behavior. This also applies in both age groups, as well as in the low and middle SES groups. When we performed regression models for each gender group, lower social support was associated with higher dietary risk behavior only in the female sample. A higher age was associated with dietary risk behavior only in the male sample. However, some studies have not found gender differences in “healthy” patterns of vegetables, legumes, fruits, cereals, potatoes, fish, and seafood among older French adults aged 65 years or older (Three-City), or vegetables, legumes, fruits, cereals, fish/seafood, and dairy products among older Quebec adults aged 67–84 years (NuAge) [33].

Partnership status and its relationship with dietary patterns has been examined a few times in the context of living situations. Therefore, living alone was positively associated with the “healthy” pattern of NutriNet-Santé [24], while living as a couple was negatively associated with the “healthy” pattern of NuAge [33]. No association could be found for the “healthy” pattern in the Three-City sample [33] and the “Mediterranean” pattern in the REACH sample [19]. In an older U.K. population aged 50 years or older, being non-partnered, living alone, and having rare/no contact with friends were negatively associated with a variety of fruits or vegetables [22]. Contrary to our expectations, we found no association between partnership status and dietary risk behavior.

In our analysis, lower SES groups consumed fewer vegetables/fruit and dairy products than higher SES groups on average. We performed regression models for each group to reveal specific differences. In the low SES group, higher alcohol intake, male gender, and less social support were associated with higher dietary risk behavior. When we analyzed educational level and income as predictors in different models, a lower educational level was associated with higher dietary risk behavior, and the same applied for both gender and age groups. Educational level was analyzed in several studies among older adults, in which a higher educational level was consistently associated with dietary patterns high in vegetables, fruit, and whole grains [24,31,33]. In the analysis of older American adults from the HRS and HCNS, income was relatively high among the “healthy” pattern [31]. However, as with the findings of Three-City and NuAge [33], we found no association between income and dietary risk behavior. Occupational status could not be analyzed due to low variance. Further research is necessary to examine the influence of occupational status and income on dietary risk behavior in older adults.

Links between the health locus of control and dietary behaviors are limited in evidence among older adults. For an Australian population aged 15–69 years, a higher internal locus of control was associated with eating vegetables and fruits regularly, as well as drinking skimmed or low-fat milk in the male sample and eating vegetables regularly in the female sample. Moreover, a higher internal locus of control was associated with physical exercise, non-smoking, and avoidance of excess drinking [36]. In our analysis, the health locus of control lost its significant effect when behavioral factors were introduced, indicating a relationship between the health locus of control and behavioral factors. However, there is a lack of studies showing the relationship between the health locus of control and dietary behavior, even in the elderly. Further studies should investigate the four individual dimensions of the health locus of control and their influence on the dietary behavior of older adults in more detail. Potential relationships could have been hidden by the unidimensional scaling or by other variables.

In an American adult sample of university and health center employees aged 18 years or older, social support was positively associated with a dietary pattern high in vegetables, fruit, and whole grains, among others [38]. This fits with our results, as less social support was associated with higher dietary risk behavior. In addition, a positive association between social support and physical activity was found in a systematic review of older adults aged 50 years or older [39]. Social support may affect dietary risk behavior not only directly, but also indirectly through behavioral factors. Overall, research suggests an association between social support and dietary behavior, yet the relationship has not been fully investigated in the older population.

Recent studies have mostly revealed a connection between smoking status and dietary behavior in older adults. The “healthy” pattern from the NuAge sample was negatively associated with smoking, whereas analysis of the “healthy” pattern from the Three-City sample showed no significant associations [33]. Older American adults with a “healthy” pattern were more likely to be non-smokers (HRS and HCNS) [31]. The “healthy” pattern of older French adults in the NutriNet-Santé cohort was positively associated with former smokers in the female sample and negatively associated with current smokers in the male sample [24]. This is in line with our finding that smokers were more likely to engage in dietary risk behavior, even though the proportion of smokers was relatively low in our sample at 6%.

Relationships between alcohol consumption and dietary behaviors have been studied less, likely because alcoholic beverages are often included in dietary patterns as a food group [19]. Nevertheless, analyses on single food groups provided trends. Thus, findings in older Ghanaian adults aged 50 years or older indicated a positive association between alcohol consumption and inadequate vegetable and fruit intake (less than five servings a day) [40]. Among older European adults aged 60 years or older, non-drinkers had the highest vegetable and fruit intake [41]. In a male sample of older Finnish adults aged 53–73 years, moderate drinkers had lower fruit intake and heavy drinkers had lower milk intake than non-drinkers. For both genders, no associations between alcohol consumption and vegetables, fruit, whole grain, and dairy products were found [42]. In the “Mediterranean” pattern from the REACH study, alcohol intake had no associations, while high physical activity was positively associated with the dietary pattern [19]. Older American adults of the “healthy” pattern from the HRS and HCNS reported regular vigorous activity more often than those with other dietary patterns [31]. In the EPIC study, the “vegetable-based” pattern was positively associated with physical activity [30]. In our analysis, associations of higher alcohol intake and lower physical activity with higher dietary risk behavior were strong.

To date, most studies have focused on identifying and comparing different dietary patterns, such as “healthy,” “Western,” “prudent,” and “Mediterranean.” Therefore, associations between social factors and dietary patterns have been investigated, and the underlying structure of the social factors has usually not been apparent. A more structured introduction of the social factors would be desirable to better compare the results with other studies.

Unfortunately, in many studies, (adjusted) *R*^2^ values were not reported, meaning that the goodness-of-fit of the models could not be assessed. Thus, it is not possible to clarify to what extent the independent variables are suited to explain variances in dietary patterns [19].

In addition, there is a lack of analyses that have examined the dietary patterns for different gender, age, and SES groups. This is a crucial issue, as an understanding of the relationship between social factors and dietary risk behavior in different social groups is needed to address specific social circumstances when developing demand-orientated dietary interventions for older adults.

### 4.4. Implications for Policy and Practice

A major goal for policy and practice should be to reduce inequity in dietary risk behavior among older adults. Our findings provide prospects for developing demand-orientated dietary interventions in older adults that may have been underestimated thus far. The results can be used to derive risk profiles encompassing target groups and fields of action, which hold significant potential for dietary interventions. For example, men with less physical activity, low SES, and higher alcohol use may need and benefit from specific interventions. However, the target groups that would most likely benefit from interventions are frequently overlooked [68,69]. Due to differences in the accessibility, utilization, and acceptability of interventions, higher SES groups often benefit disproportionately more than lower SES groups (inverse equity hypothesis) [70].

Interventions that offered free food [71] and/or short theory-based nutrition education [72] increased vegetable and fruit intake among older adults. These could be approaches to address low SES groups with limited financial resources and low educational levels [73]. However, the long-term efficacy of such intense interventions over a specific period is largely unknown [71]. In addition, older adults’ dietary risk behavior appears to be mitigated by social care and social aspects of food access. Shared meals at community centers [74] or homes [75] could benefit older adults who are not socially integrated. For older adults with less social support and a lower internal health locus of control, increasing the amount or frequency of intervention contact may be helpful for successful dietary changes [76]. Meal delivery services can reach older adults who are unable to shop for groceries or cook [77]. A systematic review revealed that meal delivery services improved diet quality, increased nutrient intake, and reduced food insecurity and nutritional risk in older adults. Other positive aspects include more opportunities for social contact and better adherence to dietary habits [78]. As physical activity, smoking, and alcohol consumption are related to dietary risk behavior, it may be helpful to combine target groups for behavioral interventions. This is already being successfully implemented in interventions that positively influence older adults’ physical activity and dietary behavior [79,80]. However, interventions also depend on the desire or readiness of individuals to change their dietary behavior [80].

On a more general level, the specific demands of older adults should be considered when planning and implementing diet-related interventions. Nutrition screening and analysis of dietary behavior are useful to identify needs and direct limited resources to those who need them most [81]. Particularly in community settings, stakeholder groups should be involved in participatory development processes to take advantage of synergetic effects and increase the acceptance of interventions [82].

## 5. Conclusions

The present analysis provided up-to-date information about the dietary risk behavior of older German adults, highlighting group differences stratified by gender, age, and socioeconomic status. Physical activity, female gender, socioeconomic status, social support, and age (in the male sample) were negatively associated with dietary risk behavior. Alcohol consumption and smoking were positively associated with dietary risk behavior. Group-specific analysis revealed higher goodness-of-fit for the low SES group, the age group of 65–79 years, and women. Our results may be transferable to interventions in other municipalities in high-income countries but should also be validated in further studies. Future studies could augment the findings by replicating the models in longitudinal, nationwide, and/or international contexts.

## Figures and Tables

**Table 1:** Characteristics of the participants.

Variables	Valid Values	Missing Values (%)	Mn	Mx	M	SD
Age (in years)	1635	3.1	65	98	76.4	6.3
Gender ^b^	1661	1.5	0	1	0.52 ^a^	0.5
Partnership status ^c^	1661	1.5	0	1	0.72 ^a^	0.5
Educational level	1666	1.2	1	7	4.1	3.3
Income	1593	5.6	1	7	4.0	1.9
Health locus of control	1624	3.7	−3	3	0.2	0.8
Social support	1647	2.4	3	14	9.7	2.0
Smoking status ^d^	1674	0.8	0	1	0.06 ^a^	0.2
Alcohol use	1516	10.1	0	11	2.9	1.9
Physical activity	1628	3.5	2	12	8.2	2.4
Dietary risk behavior	1567	1.2	−2	3	0.0	1.0

Mn: minimum; Mx: maximum; M: mean; SD: standard deviation. ^a^ Mean can be interpreted as a percentage of the distribution. ^b^ Reference group is male. ^c^ Reference group is no partnership. ^d^ Reference group is non-smoker.

**Table 2:** Consumption frequencies of vegetables/fruit, whole grains, and dairy products.

Food Groups	Valid	Several Times/Day	Once/Day	Several Times/Week	Once/Week	Rarely	Never
	*n*	*n*	*n*	*n*	*n*	*n*	*n*
	(%)	(%)	(%)	(%)	(%)	(%)	(%)
Vegetables/fruit	1648	552	698	346	33	17	2
	(98)	(34)	(42)	(21)	(2)	(1)	(0.1)
Whole grains	1600	148	494	445	148	315	50
	(95)	(9)	(31)	(28)	(9)	(20)	(3)
Dairy products	1620	313	724	424	55	77	27
	(96)	(19)	(45)	(26)	(3)	(4.8)	(2)

How often do you usually consume the following food groups? (never = 1, rarely = 2, once a week = 3, several times a week = 4, once a day = 5, and several times a day = 6). *n*: valid values; %: percentage.

**Table 3:** Dietary risk factors stratified by gender, age, and socioeconomic status groups.

	Gender			Age			Socioeconomic Status			
	Male	Female		65–79	80–98		Low	Middle	High	
	*n*	*n*	*p*	*n*	*n*	*p*	*n*	*n*	*n*	*p*
	(%)	(%)		(%)	(%)		(%)	(%)	(%)	
Valid values	785	860		1110	511		296	928	304	
Dietary risk low in vegetables/fruit	595	499	<0.001	729	353	0.18	205	617	185	0.08
	(76)	(58)		(66)	(69)		(69)	(67)	(61)	
Valid values	771	829		1086	490		285	901	304	
Dietary risk low in whole grains	712	740	0.03	985	446	0.84	266	812	275	0.26
	(92)	(89)		(91)	(91)		(93)	(90)	(91)	
Valid values	775	845		1095	498		287	914	302	
Dietary risk low in dairy products	673	634	<0.001	889	395	0.38	241	740	230	0.05
	(87)	(75)		(81)	(79)		(84)	(81)	(76)	

*n*: valid values; %: percentage; *p*: *p*-value.

**Table 4:** Differences in the consumption frequencies of single food groups stratified by gender, age, and socioeconomic status groups.

	Gender			Age			Socioeconomic Status			
	Male	Female		65–79	80–98		Low	Middle	High	
	M	M	*p*	M	M	*p*	M	M	M	*p*
	(SD)	(SD)		(SD)	(SD)		(SD)	(SD)	(SD)	
Vegetables/fruit	4.9	5.2	<0.001	5.1	5.0	0.27	4.9	5.1	5.2	0.001
	(0.9)	(0.8)		(0.8)	(0.9)		(1.0)	(0.8)	(0.8)	
Whole grain products	3.8	4.0	<0.001	3.9	3.9	0.80	3.8	4.0	4.0	0.24
	(1.4)	(1.4)		(1.3)	(1.4)		(1.4)	(1.3)	(1.3)	
Dairy products	4.5	4.8	<0.001	4.7	4.7	0.94	4.5	4.7	4.8	0.003
	(1.1)	(1.1)		(1.1)	(1.1)		(1.1)	(1.1)	(1.0)	
Valid values (*n*)	791	870		1116	519		309	931	307	

How often do you usually consume the following food groups? (never = 1, rarely = 2, once a week = 3, several times a week = 4, once a day = 5, and several times a day = 6.) M: mean; SD: standard deviation; *p*: *p*-value.

**Table 5:** Component matrix of the Dietary Risk Behavior Index.

Components	Loadings
Vegetables/fruit	0.70
Whole grain products	0.74
Dairy products	0.72

*n* = 1567; extraction method: principal component analysis.

**Table 6:** Multiple linear regression analysis of the relationship between social factors and dietary risk behavior.

Model	M 1		M 2		M 3		M 4	
	ß	*p*	ß	*p*	ß	*p*	ß	*p*
Sociodemographic factors								
Age	−0.01	0.74	−0.03	0.18	−0.05	0.06	−0.04	0.10
Gender ^a^	−0.20	<0.001	−0.27	<0.001	−0.28	<0.001	−0.22	<0.001
Partnership status ^b^	−0.03	0.34	−0.03	0.27	−0.03	0.35	−0.01	0.62
Socioeconomic factors								
Educational level			−0.20	<0.001	−0.19	<0.001	−0.17	<0.001
Income			−0.03	0.21	−0.02	0.35	−0.02	0.47
Psychosocial factors								
Health locus of control					−0.05	0.049	−0.01	0.67
Social support					−0.07	0.007	−0.06	0.01
Behavioral factors								
Smoking status ^c^							0.07	0.01
Alcohol use							0.15	<0.001
Physical activity							−0.24	<0.001
*R* ^2^	0.04		0.08		0.09		0.16	
Adj. *R*^2^	0.04		0.07		0.08		0.16	
ΔAdj. *R*^2^	0.04		0.03		0.01		0.08	
ΔF	22.61	<0.001	34.53	<0.001	7.46	<0.001	51.71	<0.001

*n* = 1687; ß: standardized regression coefficient; *p*: *p*-value. ^a^ Reference group is male. ^b^ Reference group is no partner. ^c^ Reference group is non-smoker. Adj. *R*^2^: adjusted coefficient of determination; ΔAdj. *R*^2^: adjusted coefficient of determination change; ΔF: *F*-statistic change.

**Table 7:** Multiple linear regression analysis of the relationship between social factors and dietary risk behavior stratified by gender.

Gender	Male		Female	
*n*	802		885	
	ß	*p*	ß	*p*
Sociodemographic factors				
Age	−0.09	0.02	0.00	0.90
Partnership status ^a^	−0.07	0.11	0.02	0.44
Socioeconomic factors				
Educational level	−0.17	<0.001	−0.16	<0.001
Income	−0.04	0.25	0.01	0.89
Psychosocial factors				
Health locus of control	−0.00	0.95	−0.02	0.54
Social support	−0.03	0.46	−0.09	0.003
Behavioral factors				
Smoking status ^b^	0.06	0.09	0.07	0.08
Alcohol use	0.15	<0.001	0.16	<0.001
Physical activity	−0.19	<0.001	−0.28	<0.001
*R* ^2^	0.13		0.15	
Adj. *R*^2^	0.12		0.14	

*n*: valid values; ß: standardized regression coefficient; *p*: *p*-value. ^a^ Reference group is no partner. ^b^ Reference group is non-smoker. Adj. *R*^2^: adjusted coefficient of determination.

**Table 8:** Multiple linear regression analysis of the relationship between social factors and dietary risk behavior stratified by age groups.

Age	65–79 Years		80–98 Years	
*n*	1141		546	
	ß	*p*	ß	*p*
Sociodemographic factors				
Gender ^a^	−0.24	<0.001	−0.17	0.002
Partnership status ^b^	−0.02	0.57	0.01	0.78
Socioeconomic factors				
Educational level	−0.19	<0.001	−0.12	0.01
Income	−0.01	0.79	−0.04	0.42
Psychosocial factors				
Health locus of control	0.02	0.62	−0.06	0.20
Social support	−0.08	0.007	−0.03	0.53
Behavioral factors				
Smoking status ^c^	0.07	0.01	0.06	0.55
Alcohol use	0.16	<0.001	0.13	0.01
Physical activity	−0.25	<0.001	−0.21	<0.001
*R* ^2^	0.18		0.12	
Adj. *R*^2^	0.18		0.11	

*n*: valid values; ß: standardized regression coefficient; *p*: *p*-value. ^a^ Reference group is male. ^b^ Reference group is no partner. ^c^ Reference group is non-smoker. Adj. *R*^2^: adjusted coefficient of determination.

**Table 9:** Multiple linear regression analysis of the relationship social factors and dietary risk behavior stratified by socioeconomic status groups.

SES	Low		Middle		High	
*n*	348		966		373	
	ß	*p*	ß	*p*	ß	*p*
Sociodemographic factors						
Age	−0.07	0.29	−0.00	0.99	−0.08	0.25
Gender ^a^	−0.17	0.03	−0.21	<0.001	−0.13	0.14
Partnership status ^b^	−0.01	0.91	0.00	0.95	−0.03	0.58
Psychosocial factors						
Health locus of control	−0.02	0.76	−0.01	0.79	−0.04	0.47
Social support	−0.13	0.04	−0.04	0.22	−0.08	0.14
Behavioral factors						
Smoking status ^c^	0.11	0.06	0.05	0.17	0.07	0.34
Alcohol use	0.25	<0.001	0.12	0.001	0.09	0.09
Physical activity	−0.33	<0.001	−0.22	<0.001	−0.21	<0.001
*R* ^2^	0.25		0.13		0.11	
Adj. *R*^2^	0.23		0.12		0.09	

*n*: valid values; ß: standardized regression coefficient; *p*: *p*-value. ^a^ Reference group is male. ^b^ Reference group is no partner. ^c^ Reference group is non-smoker. Adj. *R*^2^: adjusted coefficient of determination.

## Data Availability

The data presented in this study are available on request from the corresponding author. The data are not publicly available due to restrictions imposed by the Puchheim Municipal Administration.
